# Gender plays no role in student ability to perform on computer-based examinations

**DOI:** 10.1186/1472-6920-6-57

**Published:** 2006-11-28

**Authors:** Susan M Kies, Benjamin D Williams, Gregory G Freund

**Affiliations:** 1 190 Medical Sciences Building, MC-714, 506 South Mathews Avenue, Urbana, IL 61801, US

## Abstract

**Background:**

To see if there is a difference in performance when students switch from traditional paper-and-pencil examinations to computer-based examinations, and to determine whether there are gender differences in student performance in these two examination formats.

**Methods:**

This study involved first year medical students at the University of Illinois at Urbana-Champaign over three Academic Years 2002–03/2003–04 and 2003–05. Comparisons of student performance by overall class and gender were made. Specific comparisons within courses that utilized both the paper-and-pencil and computer formats were analyzed.

**Results:**

Overall performance scores for students among the various Academic Years revealed no differences between exams given in the traditional pen-and-paper and computer formats. Further, when we looked specifically for gender differences in performance between these two testing formats, we found none.

**Conclusion:**

The format for examinations in the courses analyzed does not affect student performance. We find no evidence for gender differences in performance on exams on pen-and-paper or computer-based exams.

## Background

Delivery of examinations via computer (on-line testing) is becoming more and more prevalent in medical education. Since 1998, students have taken the United States Medical Licensing Examination (USMLE) Step 1, Step 2 and Step 3 on-line. In the very near future, the Medical College Admissions Test (MCAT) will be administered on-line only. The National Board of Medical Educators is now developing on-line Subject Examinations. Current thinking is that on-line testing is the same as paper-and-pencil administration [1-5].

Given the sweeping changes that are now occurring in medical school testing methods, it is important to understand the potential performance differences in students taking paper-and-pencil examinations compared to on-line examinations and to implement on-line examinations so that students have the best opportunity to show their level of proficiency [6,7].

This study involves first year medical students at the University of Illinois at Urbana-Champaign. During Academic Year 2004–05 the biochemistry and neuroscience courses each began administering one of their major examinations via computer. The purpose of the study was to detect any differences in student achievement between traditional paper-and-pencil and computer-based exams, and furthermore, to detect any gender-based differences in student achievement using these different testing modalities.

We decided include an analysis of gender on our study because of several studies over the past thirteen years suggest that there is a 'technological gender gap' between males and females, with female subjects falling behind their male peers in use of computers [8-13]. In 1992 Canada and Brusca found that females tend to perceive themselves as less equipped to deal with computers. Eleven years later, in 2003, Lee reported results from surveys of college-age students regarding computer use. He found that, "...nearly half of females respondents...rated themselves as someone with 'limited experience' compared to slightly more than one-third of the males (in regard to computer use.)" Interestingly, Fallows found that as students progress in their studies the male/female technology gap appears to widen. Although more recent studies suggest that females have made some gains in computer use, they nevertheless conclude that in many significant ways females lag behind males in their use of computers and specifically Internet use [8].

The authors hypothesized students would perform less well on computer-based exams than on traditional pen-and-paper exams, and that much of this difference would reflect poorer performance by females on computer-based exams.

## Methods

During Academic Year 2004–2005 178 students took M-1 examinations at the University of Illinois College of Medicine at Urbana-Champaign. The questions consisted primarily of Multiple Choice Questions (MCQ), most examinations were traditional paper-and-pencil tests, with students marking on the examination booklet and ultimately making their answer choice on a bubble scan sheet. For computer-based examinations, students selected their answer choice by clicking a web-page "radio button." It is important to note that during all computer-based examinations, students are given a scratch paper for notes. Also the on-line examinations were designed according to the best practices as outlined in the literature [2,7].

M-1 courses in this study were categorized according to the definitions in Table 1. 'P&P' indicates courses in which all course examinations, including mid-term and comprehensive final exams, were in the paper-and-pencil format during all three years of the study. 'Mixed' indicates courses in which the comprehensive final examination was given in the traditional format in year one and two, but then changed to the on-line format in the third year of the study.

Table 1 also describes the analyses performed in the study. It is broken into two sections that describe the metric used to analyze data comparing Academic Year performance over years 1, 2 and 3. The section outlines the analysis of student performance within the disciplines (courses) that employ a mix of on-line and paper-and-pencil examinations. The control discipline only employed paper-and-pencil examinations to measure student performance.

After Institutional Review Board approval, the population studied included all students enrolled in biochemistry, medical statistics and neuroscience in the first year medical school curriculum at the University of Illinois College of Medicine at Urbana-Champaign.

### Data analysis

Data analysis was performed in several steps by analyzing class performance in all M-1 courses over three Academic Years: 2002–03 (year 1); 2003–04 (year 2); and, 2004–05 (year 3). The first step was to determine if general M-1 performance differences could be caused by differences in class ability. Because there were three factors (class year, gender and test type) that could potentially affect student performance as represented by final mean score, a multi-factorial ANOVA was employed to examine simultaneously the effects of these factors on score and to assess whether they are having a significant impact and also whether possible interactions between these factors are having significant effects. Then, within each discipline in the study, a multi-factor ANOVA was employed to determine the influence these factors (gender, paper-and-pencil exams or on-line exam) had on the overall performance within each discipline. Then, within each course, gender differences related to performance were studied. Specifically individual final mean scores during each of the three years were compared. These data were analyzed employing SPSS 14.

## Results

### Analysis of overall performance by Academic Year

First, the multi-factor ANOVA shows that there was no significant difference in the performance of students comparing Academic Years 2002–03; 2003–04; and, 2004–05. The male and female performance are equal in all years as is the scores on paper versus computer exams reveal no difference. There appears to be no significant interaction between these factors.

### Analysis of performance within each discipline

#### Paper-and-Pencil (P&P) course

The P&P (Medical Statistics) course was used as a control. ANOVA comparisons of final grades in Medical Statistics were made to see if there were differences in performance over the Academic Years in the study. (AY 03 n = 126, AY 04 n = 131, AY 05 n = 127) ANOVA comparisons revealed no differences in overall performance over AY 2002–03; 2003–04; and, 2004–05. (See Table 2.)

#### Mixed courses

A multi-factor ANOVA, comparing the test format (paper-and-pencil or on-line exam) performance, the gender and final score was analyzed. The results revealed no differences in performance by gender. Further, no significant interaction between these factors was found. (See Table 3 and 4.)

If there was a difference in student performance when switching test modalities, then we expected to see changes in performance in the Mixed Courses. First we examined the overall course performance of students and influence of the paper-and-pencil exams, the on-line exams and the gender of the subjects. Results of analysis for mixed courses revealed a no difference in class performance in Biochemistry, nor in Neuroscience based on gender or examination type. (See Table 5.)

## Discussion

Conceived and created by Benjamin Williams, Ph.D. in Academic Year 1999–00, the University of Illinois College of Medicine at Urbana-Champaign has carefully and methodically developed a computer based testing software package originally designed to test images in the histology course for first year medical students. Since 1999 histology students have taken their final examinations on-line using the software developed by Dr. Williams. During the past two years, a significant effort to expand the software has resulted in a flexible, powerful and secure program that completely manages all aspects of assessment in the first year medical curriculum in Urbana. The on-line examinations were designed taking into consideration best practice as outlined in the literature [2,7].

Further, all students become very well acquainted with the computerized format long before taking examinations through the use of on-line practice examinations and on-line grade reporting. Thus, giving all students, and especially females, a level of comfort and familiarity with the on-line format long before an on-line examination is administered.

During Academic Year 2004–05 faculty in biochemistry and neuroscience elected to have their final examinations administered in the new on-line format. The purpose of this study was to determine if students were placed at a disadvantage when taking on-line examinations in lieu of paper-and-pencil examinations and to determine if gender played a role in performance.

Analysis revealed that no difference in overall performance related to gender was found.

No difference in overall class performance was found, closer examination revealed no difference in performance when comparing males and females, regardless of the test format and regardless of discipline. Both the multi-factor ANOVA and the specific t-Tests, performed within each discipline revealed no performance difference between males and females in either one-tail or two-tail distributions.

### Neuroscience

Neuroscience is a single-semester course delivered during the Spring Semester during the M-1 curriculum. There are two examinations, one in March and one in May. During Academic Year 2004–05, the May examination was administered in an on-line format. When comparisons were made over three academic years of overall student performance, no performance differences were found. However, there were interesting differences between the classes' performance on both the paper-and-pencil comparisons and the paper-and-pencil comparisons to on-line format. Students performed better with the on-line administration of the exam. It should be noted that increases in performance are typically demonstrated on the final examination in this course, so it is not surprising to find a difference in performance as most students attempt to pass this course.

### Biochemistry

Biochemistry is a semester and one-half course delivered from August until March during the M-1 curriculum. There are four examinations, three during the Fall Semester and one during the Spring Semester. During the Spring Semester the format changes from a traditional lecture format to small group application of biochemistry principles to patient cases. The fall examinations are administered in traditional paper-and-pencil format. The March examination, which is a combination of short-answer and multiple choice questions switched from paper-and-pencil to on-line format during Academic Year 2004–05. ANOVA comparisons of student performance during Academic Years 02, 03 and 04 revealed significant differences in overall performance in the course. ANOVA comparisons on the on-line examination also revealed differences in performance. Similar to the neuroscience performance, it is difficult to draw the conclusion that performance differences between the paper-and-pencil compared to on-line are a result of change in format. These changes could be due to differences in grading of the short answer questions and the change from hand-written responses to word-processed responses.

With regard to gender performance differences in biochemistry, analysis revealed none.

The lack of performance differences in medical students relative to gender is could be explained by the characteristic profile of this population. Regardless of gender, medical students are hardworking, well-informed and technology-capable. Perhaps the general population of females, as opposed to the medical student population of females, is at a greater risk when it comes to utilization of technology in an assessment setting. Further studies designed to address this issue should be made at all levels of the educational/training experience.

## Conclusion

Despite overwhelming evidence in the literature regarding gender differences in computer use and attitude toward computer use, females are not placed at a disadvantage when administered on-line examinations at the University of Illinois College of Medicine at Urbana-Champaign. M-1 students do not perform more poorly on on-line examinations compared to traditional paper-and-pencil examinations.

As the formats of course examinations and standardized examinations change from paper-and-pencil to the on-line format, it is imperative that testing design pay close attention to the best practice suggestions outlined in the literature and give students an opportunity to familiarize themselves with the test delivery software.

## Abbreviations

None

## Competing interests

The author(s) declare that they have no competing interests.

## Authors' contributions

SMK conceived of the idea, carried out the analysis and wrote the initial draft. BDW and GGF assisted in developing the manuscript, consulted on the analysis and approved the final manuscript. All authors read and approved the final manuscript.

## Pre-publication history

The pre-publication history for this paper can be accessed here:



## Figures and Tables

**Table 1:** Courses Included in the Study

Category Name	Category Definition	Course(s)	
P&P	All paper-and-pencil examinations	Medical Statistics	
Mixed	Combination of written examinations and on-line final in Academic Year 2004–05	Neurosciences, Biochemistry	
**Analysis of Overall Performance by Academic Year**			

Name of Study	To Whom the Analysis was Applied	Metric Employed	Course Category

Overall Performance	All Students' Mean Score over all courses During AY 2002–03; 2003–04; and, 2004–05	ANOVA	All
**Analysis of Performance within each Discipline**			

Name of Study	To Whom the Analysis was Applied	Metric Employed	Course Category

Overall Performance	All Students' Mean Score over AY 2004–05 Within the course comparing gender, paper and On-line exam performance	ANOVA	Mixed P&P
Gender	All Students' Mean Score over AY 2004–05 Within the course comparing gender.	t-Test	Mixed

**Table 2:** This table shows that there is no difference between the three factors of interest which may affect performance as represented by score. They are gender, year, then gender within year and the dependent variable is mean score or year-end performance. These include data for all courses during Academic Years 2002–03; 2003–04; and, 2004–05.

**Tests of Between-Subjects Effects**								
Source		Type III Sum of Squares	df	Mean Square	F	Sig.	Noncent. Parameter	Observed Power(a)
Intercept	Hypothesis	1654056.805	1	1654056.805	16592.664	.000	16592.664	1.000
	Error	199.459	2.001	99.686(b)				
gender	Hypothesis	211.286	1	211.286	3.535	.201	3.535	.201
	Error	119.612	2.001	59.763(c)				
Year	Hypothesis	199.402	2	99.701	1.668	.375	3.337	.126
	Error	119.526	2	59.763(d)				
gender *	Hypothesis	119.526	2	59.763	1.012	.365	2.024	.226
Year	Error	17949.374	304	59.044(e)				

Dependent Variable: Mean Score End of Year

Computed using alpha = .05

**Table 3:** ANOVAs examining the influence gender and examination type (paper-and-pencil or on-line) have on students' overall scores within mixed disciplines. This analysis shows no difference in gender performance nor on type of examination.

**Tests of Between-Subjects Effects**					
**Biochemistry**					
Source	Type III Sum of Squares	df	Mean Square	F	Sig.
Corrected Model	6141.833(a)	94	65.339	32.669	.139
Intercept	335684.810	1	335684.810	167842.405	.002
gender	24.500	1	24.500	12.250	.177
paper	306.440	15	20.429	10.215	.241
computer	1743.435	51	34.185	17.093	.190
gender * paper	.000	0	.	.	.
gender * computer	.000	0	.	.	.
paper * computer	42.667	2	21.333	10.667	.212
gender * paper * computer	.000	0	.	.	.
Error	2.000	1	2.000		
Total	420632.000	96			
Corrected Total	6143.833	95			

Dependent Variable: score

a R Squared = 1.000 (Adjusted R Squared = .969)

**Table 4:** Neuroscience: Tests of Between-Subjects Effects: This analysis shows a difference in student performance based on type of examination.

Source	Type III Sum of Squares	df	Mean Square	F	Sig.
Corrected Model	7013.661(a)	46	152.471	107.991	.000
Intercept	173513.949	1	173513.949	122895.618	.000
computer	3137.736	26	120.682	85.476	.000
paper	835.220	19	43.959	31.135	.000
gender	.034	1	.034	.024	.877
Error	67.770	48	1.412		
Total	533536.000	95			
Corrected Total	7081.432	94			

Dependent Variable: final score

a R Squared = .990 (Adjusted R Squared = .981)

**Table 5:** t-Test reveals no differences between male and female performance on the on-line final in Biochemistry, Neuroscience and Medical Statistics Independent Samples Test

		Levene's Test for Equality of Variances		t-test for Equality of Means						
		
									95% Confidence Interval of the Difference	
									
		F	Sig.	t	df	Sig. (2-tailed)	Mean Difference	Std. Error Difference	Lower	Upper
biochem4	Equal variances assumed	3.088	.081	-.207	130	.836	-.40005	1.93137	-4.22104	3.42095
	Equal variances not assumed			-.208	127.627	.835	-.40005	1.91983	-4.19886	3.39877
neurofinal	Equal variances assumed	.041	.839	-1.507	123	.134	-2.57732	1.71061	-5.96336	.80873
	Equal variances not assumed			-1.508	122.247	.134	-2.57732	1.70930	-5.96099	.80635
stats	Equal variances assumed	.000	.994	.794	124	.429	1.07007	1.34748	-1.59696	3.73711
	Equal variances not assumed			.793	121.237	.429	1.07007	1.34944	-1.60145	3.74160
